# Maternal Parenting Stress in the Face of Early Regulatory Disorders in Infancy: A Machine Learning Approach to Identify What Matters Most

**DOI:** 10.3389/fpsyt.2021.663285

**Published:** 2021-08-02

**Authors:** Anna K. Georg, Paul Schröder-Pfeifer, Manfred Cierpka, Svenja Taubner

**Affiliations:** ^1^Institute for Psychosocial Prevention, Centre for Psychosocial Medicine, Heidelberg University Hospital, Heidelberg, Germany; ^2^Psychological Institute, University Heidelberg, Heidelberg, Germany

**Keywords:** early regulatory disorders, machine learning algorithms, parenting stress, parental self-efficacy, infant mental health diagnostic

## Abstract

**Objective:** Early regulatory disorders (ERD) in infancy are typically associated with high parenting stress (PS). Theoretical and empirical literature suggests a wide range of factors that may contribute to PS related to ERD. The aim of this study was to identify key predictors of maternal PS within a large predictor data set in a sample of *N* = 135 mothers of infants diagnosed with ERD.

**Methods:** We used machine learning to identify relevant predictors. Maternal PS was assessed with the Parenting Stress Index. The multivariate dataset assessed cross-sectionally consisted of 464 self-reported and clinically rated variables covering mother-reported psychological distress, maternal self-efficacy, parental reflective functioning, socio-demographics, each parent's history of illness, recent significant life events, former miscarriage/abortion, pregnancy, obstetric history, infants' medical history, development, and social environment. Variables were drawn from behavioral diaries on regulatory symptoms and parental co-regulative behavior as well as a clinical interview which was utilized to diagnose ERD and to assess clinically rated regulatory symptoms, quality of parent–infant relationship, organic/biological and psychosocial risks, and social–emotional functioning.

**Results:** The final prediction model identified 11 important variables summing up to the areas maternal self-efficacy, psychological distress (particularly depression and anger-hostility), infant regulatory symptoms (particularly duration of fussing/crying), and age-appropriate physical development. The RMSE (i.e., prediction accuracy) of the final model applied to the test set was 21.72 (*R*^2^ = 0.58).

**Conclusions:** This study suggests that among behavioral, environmental, developmental, parent–infant relationship, and mental health variables, a mother's higher self-efficacy, psychological distress symptoms particularly depression and anger symptoms, symptoms in the child particularly fussing/crying symptoms, and age-inappropriate physical development are associated with higher maternal PS. With these factors identified, clinicians may more efficiently assess a mother's PS related to ERD in a low-risk help-seeking sample.

## Introduction

Early regulatory disorders (ERD), which include sensory, sleeping, crying, or feeding disorders, are found in 10.9% of infants/toddlers and are among the most prevalent diagnoses in children under the age of four (1). The disorders have been repeatedly found to be associated with high parenting stress (PS) and parental burden (2, 3). Research has primarily focused on the effects of excessive crying and infant colic on parents: e.g., compared to control groups, mothers reported higher negative affect in response to the cries (4) and felt more sad and aroused by the cries (5). According to the developmental system model of ERD, the parental stress response to infants' regulation problems may contribute to a vicious circle of negative contingency that perpetuates parental burden, impairs parental self-efficacy, and leads—in the context of parent and child-related risk and protective factors—to the manifestation or perpetuation of ERD (6). Contrarily, in the context of a lower distress response of parents and through being more effectively co-regulated, infants' self-regulatory competence may increase.

Research showed that while ERD likely have far-reaching consequences for a child (7), they do not necessarily have such effects, but may be mediated by parents' burden. Smarius et al. found that the maternal burden of infant care partially mediated the association between ERD and later mood and behavioral problems in childhood (8). In addition, the level of maternal PS predicted the persistence of regulation problems (9). These studies suggest that reducing PS may be an effective objective in treating ERD and may also contribute to better long-term outcomes.

While the adverse effects of ERD on parents have been well-established, the specific risk and protective factors associated with PS when raising infants with ERD have yet to be explored. A set of risk factors for ERD have been proposed (6, 10), many of whom may play a role in parents' propensity to experience PS related to ERD: high prenatal maternal stress or lifetime depressive or anxiety disorders have been found to predict ERD (11, 12), and socio-demographic risk factors, such as low social support or low maternal education, have been shown to be related to ERD (9, 11, 13) and may negatively affect PS (14, 15). PS has also been linked to miscarriages or abortions (15), which have been found to be more prevalent in a clinical ERD sample (6). Peripartum risk factors, like complicated pregnancy or birth which are more frequent in ERD samples (6, 16), may affect parents' perception of infants' distress and thus their propensity to experience PS. Other infant diagnostic characteristics, such as the presence of an organic condition or difficult temperament, were related to ERD (6, 13) and may increase PS (17). Maternal self-efficacy was a protective factor for reporting ERD (11, 18) and may protect against high PS. Similarly, parental reflective functioning may be a protective factor for high PS related to ERD (19).

While the existing body of literature provides valuable data on distinct predictors and correlates of ERD, the extent to which these variables are associated with maternal PS (MPS) in the context of ERD has rarely been investigated. An additional limitation of the literature is the inclusion of a small number of variables, despite multiple and interrelated factors within a family system that may relate to PS. Thus, clinicians wishing to help families who experience ERD by reducing parental burden of infant care must assess a vast number of possible variables that may be related to the PS. Furthermore, few of the studies included samples of infants with ERD beyond excessive crying. However, most of the help-seeking parents report multiple regulatory problems (3, 13) and there is a need to understand more about this group. Thus, the goal of this study was to identify key variables associated with MPS in ERD by utilizing a large predictor variable set. To this end, machine learning (ML) was applied.

ML approaches for clinical psychology and psychiatry perform statistical functions on multidimensional data sets to make generalizable predictions about individuals, e.g., they can be used to provide predictive models for diagnostic classification or treatment response of individual cases. In the field of child psychiatry, ML may prove especially useful for the incorporation of data from different sources like behavior measures, genetics, and environmental as well as developmental factors (20). This advantage is a product of ML being able to integrate large sets of correlated variables while assuming no distribution in the outcome or underlying data mechanism and being largely insensitive to outliers (21). In addition, prediction models can include data on single-item level. For example, Carpenter et al. tested whether individual diagnostic interview items from the Preschool Age Psychiatric Assessment predicted with high reliability and low false-positive rates whether a child is likely to have an anxiety disorder (22). The resulting variable importance ranking can be utilized to shorten diagnostic batteries for more effective diagnostics and for identifying cases that may need further evaluations. All of these features are especially useful for the field of infant mental health research where multiaxial diagnostics are the norm.

We employed ML in an exploratory search for variables best predicting a mother's MPS related to ERD in a cross-sectional study design. Predictor variables were empirically and theoretically derived (6, 10) and covered risk and protective factors as well as correlates that have been identified for ERD or PS. We included a multivariate dataset by utilizing multiple measures covering self-report and clinical ratings: mother-reported general psychological distress, maternal self-efficacy, parental reflective functioning, socio-demographic variables, each parent's history of illness, recent significant life events, former miscarriage/abortion, pregnancy, obstetric history, infants' medical history, development, and social environment. Behavioral diaries were used to assess infants' regulatory symptoms and extent of parental co-regulative behavior. We used all items from a structured clinical interview that was utilized to diagnose ERD and for the clinical assessment of regulatory symptoms, quality of the parent–infant relationship, organic/biological and psychosocial risks, and social-emotional functioning. In addition to global scores obtained from the instruments, we included all items gathered in our dataset on single-item and subscale levels in an effort to maximize specificity of the predictors. ML enabled us to analyze this large number of potential predictors simultaneously.

## Materials and Methods

Data were acquired from an RCT on the effectiveness of brief parent–infant psychotherapy for ERD, where data collection was still ongoing by the time of this study. We used baseline data gathered pretreatment at one time point. Data were collected from February 2014 to May 2017 in the department of Family Therapy at Heidelberg University Hospital.

The approval for research in this sample was obtained from the Ethical Committee of the Medical Faculty of Heidelberg University (approved in November 2013).

### Participants

Families were referred from pediatric practices for the purpose of study participation if parents reported significant crying, sleeping, or feeding difficulties. Some families self-referred in response to public advertisement, websites, and flyers/posters distributed in gynecological, pediatric, and osteopathic practices, parent–infant groups, and crèches. All families participated in the RCT and were randomized to treatment conditions after data collection.

Inclusion criteria required the infant to be between 4 and 15 months old, to be born at full term (>37 weeks of gestation), and to meet diagnostic criteria for sleeping disorders, feeding disorders, or regulation disorders of sensory processing according to DC:0-3 R (23) or for persistent excessive crying, sleeping, and feeding disorder, according to the guidelines recommended by the German Society of Child and Adolescent Psychiatry, Psychosomatics and Psychotherapy (AWMF guidelines; AWMF No. 028/028) (24). Pregnancy needed to be singleton, and primary caregivers needed to speak German.

Participants were excluded when infants had a medical diagnosis that better explained the regulatory problems, a tentative diagnosis of fetal alcohol syndrome, or a diagnosed disability or developmental disorder. A very high symptom severity of the primary caregiver (Symptom-Check-List-90R-S, Global Severity Index of *T* > 70) also led to exclusion (25), as a current mental illness of the caregiver was considered to be a contraindication for the brief intervention given to the families within the RCT.

A total of 165 primary caregivers expressed their interest in study participation and underwent screening for eligibility via telephone. Parents were informed about the study and invited for participation if they consented. Of these, 24 canceled or did not show up. Six families fulfilled exclusion criteria and thus were excluded. The primary caretaker was asked to participate, which in all cases was the mother. The final sample consisted of *N* = 135 mother–infant dyads.

### Procedure and Assessments

Self-report measures and behavioral diaries were mailed to mothers following the phone screen. Clinical diagnostics were led by two psychologists. The assessment was conducted with mother and infant and included the clinical interview and video recording of standardized parent–infant interactions. Written informed consent was gathered at the beginning of the session. Clinical ratings were performed immediately after the interview.

#### Maternal Parenting Stress

The Parenting Stress Index (26) assesses self-reported PS with 48 items. Items are rated on a five-point Likert scale from 1 (*strongly disagree*) to 5 (*strongly agree*). Higher scores indicate higher PS. Items are summed up into one global score. Cronbach's α was excellent in this study (0.94).

#### Psychological Distress

The Symptom-Checklist (Symptom-Checklist-90R-S, SCL) assesses self-reported psychological symptoms and psychopathology (25). The 90 items are rated on a five-point Likert scale from 0 (*not at all*) to 4 (*extremely*) with higher scores indicating higher distress. Items add up to 10 subscales (somatization, obsession–compulsion, interpersonal sensitivity, depression, anxiety, anger–hostility, phobic anxiety, paranoid ideation, psychoticism, and additional clinical symptoms), a sum score, and the global severity index (GSI). Cronbach's α was between 0.56 (psychoticism) and 0.84 (obsession–compulsion) and was excellent for the sum score (0.96).

#### Maternal Self-Efficacy

The Maternal Self-Efficacy Scale (MSES) assesses self-reported behavioral competence in parenting (27). For this study, back-translation procedures were implemented, and the final version was reviewed by an English native speaker. The 10 items are rated on a four-point Likert scale from *not good at all* (1) to *very good* (4). Cronbach's α was acceptable (0.75).

#### Parental Reflective Functioning

The Parental Reflective Functioning Questionnaire (PRFQ) uses 18 items in order to assess the scales (28): (a) interest and curiosity in mental states (IC), (b) certainty of mental states (CMS), and (c) prementalizing (PM). Items are rated on a seven-point Likert scale ranging from *strongly disagree* (1) to *strongly agree* (7). Cronbach's α was acceptable for CMS (0.73), poor for PM (0.57), and unacceptable for IC (0.47).

#### Parent-Questionnaire

The Parent-Questionnaire (29) was developed for the comprehensive data assessment of parents and their children with ERD. Questions refer to the areas socio-demographic information, history of illness, recent significant life events, former miscarriage/abortion, pregnancy, obstetric history, and infant medical history, development, and social environment. Variables are assessed dimensionally and categorically or in open format; no sum scores are provided. For the analysis, 110 single items were used (see Supplemental Material and Supplementary Table 1, which lists the items per area).

#### Clinical Interview

A structured clinical interview was developed to assess axis I (DC:0-3R) (23) on sleep-onset disorder, night-waking disorder, feeding disorders, and regulation disorders of sensory processing. Persistent excessive crying syndrome is not mentioned as a clinical category in DC:0-3R, and diagnostic criteria are poorly described (23). Therefore, we additionally utilized the AWMF guidelines on persistent excessive crying, sleep-onset disorder, night-waking disorder, feeding disorders, and pervasive regulatory disorder (AWMF) (24). The parent–infant relationship global assessment scale (PIR-GAS, DC: 0-3R) dimensionally assesses the parent–infant relationship from *documented maltreatment* (0–10) to *well adapted* (91–100). Medical conditions of the infant (axis III of DC:0-3R) and psychosocial stressors (axis IV of DC:0-3R) were dimensionally assessed using organic/biological and the psychosocial risk scales (30). Infants' emotional and social functioning (axis V of DC:0-3R) was rated on the proposed rating scale (DC:0-3R). In sum, 150 variables covering single symptoms, sum scores of symptoms on the level of diagnosis and axis, and a general symptom sum score were used in analysis (see Supplementary Table 1).

#### Infant Regulatory Symptoms

The Questionnaire for Crying, Feeding, and Sleeping (QCFS) (31) assesses crying, sleeping, and feeding symptoms and parents' dysfunctional co-regulation behavior in response to the symptoms (e.g., “only falls asleep when being carried”). The 53 items constitute the three scales, (a) fussing/crying and sleeping, (b) feeding, and (c) dysfunctional co-regulation, and a global score. Higher scores indicate more symptoms, parental burden, and dysfunctional co-regulation. Frequency questions are rated on a four-point Likert scale from *never/rarely* (1) to *always/every day* (4). Parents' perceived difficulty is rated from *not at all* (1) to *a lot* (4). Cronbach's α is good for the scales (scale 1 = 0.82; scale 2 = 0.76; scale 3 = 0.84) and the global score (0.82).

#### 96-H Behavior Diary

The diary of crying, sleeping, and feeding behavior (32) is similar to widely used parental diaries and assesses infants' behavior and parents' co-regulation behavior. Frequency and duration of fussing/crying, sleeping/waking, feeding, and parental co-regulation is recorded in 15-min intervals on four consecutive days. Additional questions refer to the success of parental co-regulative strategies. In sum, 139 variables were used in the analysis (see Supplementary Table 1).

#### Infant Development

The Ages and Stages Questionnaire (ASQ-3) is a series of 21 parent-rated questions on children's developmental performance in communication, gross motor, fine motor, problem solving, and personal–social skills, represented on five scales (33). The 30 items are rated with regard to the child's competence as *yes* (10), *sometimes* (5), or *not yet* (0). We used the German translation of the questionnaires for 4-, 6-, 8-, 9-, 10-, and 14-month-old infants. Internal consistency of the scales was not calculated, due to some small age-dependent subgroups. Other studies have shown it to be poor to excellent (33).

The PSI, SCL, and the QCFS are valid, reliable measures. For the MSES, PRFQ, and ASQ-3, validity and reliability have only been demonstrated for the original English version.

### Statistical Analysis

Since ML algorithms do not assume a certain distribution underlying the data (i.e., normal distribution, binomial distribution), the common assumptions for classical parametric analysis like homoscedastic or normally distributed residuals do not apply and the analysis handles smaller sample sizes in relation to the number of variables.

For the prediction of the PSI, all data provided by questionnaires, behavioral diary, and clinical interview on the level of items, subscales, and global scores were used, resulting in 596 variables. Of these, variables with <50% missing values before imputation were used, resulting in a final set of 464 variables. The remaining data contained 5.48% missing values. Imputation was done assuming missing at random after visual inspection of pattern of missingness plots. Multiple imputations by chained equations (34), using fully conditional specification with 40 iterations, were utilized to produce asymptotically unbiased estimations of the data.

An important difference between ML approaches and more commonly used statistical methods is the absence of *p* values and, furthermore, in-sample model fit as a measure of “success.” In ML, the main statistic of interest is the prediction accuracy which is why there are usually two phases: Training the algorithm and testing the result for generalizability. To this end, data in our study was split into a training set containing 70% of all cases and a test set containing the remaining 30%.

The general process of data analysis was carried out as follows. In the first phase, we trained our ML algorithm on the training set in order to select the best-performing algorithm. This was done by using Gradient Boosting Machines (GBM) with feature selection (FS). The algorithm was trained using 5-fold cross-validation and 10 repeats. In this procedure, the data is split into 5 folds (i.e., groups), and each fold is, in turn, left out of the training procedure and used to validate the results of training. The resulting accuracies are then averaged and provide a stable estimate of generalizability (21). In the case of the present study, the data of the 95 participants that were randomly selected to be the validation set was first divided at random into 5 folds of 19 participants. Following, the algorithm predicted the PSI value of all participants in one of the 5 folds based on the data of the other 4 folds. The difference between the predicted PSI values in that fold and the observed PSI values was then computed and averaged (in our case, root mean square error, RMSE, as well as mean absolute error MAE were calculated) over all observations. A relatively low difference between the observed and predicted values is an indicator of good generalizability, while high differences indicate bad generalizability. This process was repeated with every fold, and the result was averaged over all iterations. This was then, in turn, repeated 10 times with different splitting points to form the folds of 19 participants for the data.

In the second phase, the so trained model was validated using the holdout data of the remaining 40 participants. Prediction accuracy was computed by comparing values predicted by the trained algorithm with the observed PSI values of the holdout sample in the same manner as described above.

All statistical analyses were performed with R version 3.5.2 (35). The R package “caret” version 6.0-76 was used to train the algorithm.

#### Feature Selection (FS)

FS is a procedure to select optimal variables from a larger data set with the aim of increasing predictive performance and is part of preprocessing the data. We utilized FS since our data covered a heterogeneous set of markers and we predicted that our variable set would have few variables with large predictive effects on the PSI and many variables with medium to small effects. We used a recursive backward selection, based on importance ranking of random forests out of the entire set of 464 variables. The procedure works by fitting a random forest model using all predictors and calculating the contribution of each predictor using the variable importance metrics. Following, starting from *S* = 464 and going down to *S* = 1, a similar random forest model is fitted using the S most important variables calculated before and the performance of the model is tracked. Based on the performance profile of all models S464 through S1, the appropriate number of predictors for the final model was determined by using the predictors of the best-performing model.

The result was a set of 50 variables that were deemed to be most informative in terms of PSI and which were used in combination with the algorithm as described below. The FS “rfe” function from the caret package was used to implement this.

#### Gradient Boosting Machines (GBM)

For the final model following the feature selection, we used GBM which is suited for data with established features and is relatively easy to tune. The GBM was tuned by a hyperparameter grid search for the optimal model parameters, which was done by iteratively manipulating the shrinkage coefficient (eta) between 0.01 and 0.2, the interaction depth of each tree (max_depth) between 1 and 6, and the number of boosting iterations (nrounds) between 1 and 1500, while keeping the minimum loss reduction (gamma) fixed at 0 and the minimum sum of instance weight (min_infant_weight) fixed at 1. This represents a conservative approach with low likelihood of overfitting. The final values for the model were eta = 0.01, nrounds = 500, max_depth = 1. The gradient boosting model “xgbTree” from the caret package was used.

In an effort to rank the predictors of the PSI according to their importance or contribution for predicting PSI scores, we analyzed the variable importance of the final GBM model. This was done by summing the relevance for each predictor variable for each internal node of the tree for which the predictor was chosen as a splitting variable. Since this measure is relative, the most important variable was assigned the value of 100 while the others were scaled accordingly.

After the most relevant variables had been identified, the next step was to assess the marginal effects in which each of the predictor variables influenced PSI. Therefore, we examined partial dependency plots of the most important variables and their interrelation in predicting PSI. Marginal effects were calculated using Friedman's tree traversal method (36).

## Results

### Participants

Table 1 gives an overview of the sample characteristics. On average, the parent–infant relationship was rated as perturbed (PIR-GAS, 71-80). The percentage of maternal lifetime mental illness was lower compared to the lifetime prevalence rates in Germany (25.2%) (37). Mothers' average psychological distress (SCL-GSI) was equivalent to a *T*-score of 57, which is ~>1 *SD* higher compared to the normative sample (25). On average, they experienced more MPS (PSI) than 88% of the normative sample (26).

**Table 1:** Sample characteristics of infants and their mothers (*N* = 135).

**Variable**	***M*/%**	***SD***
Infant age (in months)	8.55	3.10
Mother age (in years)	33.27	4.47
Girls	45.2%	–
Firstborn child	65.2%	–
Mother has high school or higher education	74.8%	–
Mother married	79.3%	–
Mother of German origin	79.3%	
Mother with mental disorder lifetime	14.8%	–
**Diagnoses**		
Persistent excessive crying	8.9%	–
Regulation disorder of sensory processing	44.4%	–
Feeding disorder	13.3%	–
Sleeping disorder	95.6%	–
> 1 diagnoses	48.0%	–
PIR-GAS	74.96	9.76
SCL (GSI)	49.00	34.18
PSI	131.50	31.60

*PIR-GAS, Parent–Infant Relationship General Assessment; SCL (GSI), Global Severity Index of the Symptom-Severity-Check-List-90R-S; PSI, Sum score of the Parenting Stress Index*.

### Performance

The RMSE (i.e., prediction accuracy) of the final model applied to the test set was 21.72, the *R*^2^ was 0.58, and the MAE was 17.04. Thus, the algorithm on average over- or underestimated the observed PSI score of the participants by 17.04 points or within 10.72% of the observed PSI range which was 159. Thus, by using the final model, an individual mothers' MPS can be predicted within the range of 17.04 points on the PSI score which equals about half a standard deviation on the observed PSI range.

The relatively small difference between RMSE and MAE indicates that there were few observations that had larger than average residuals. This indicates that the model's predictive performance is independent of the observed PSI scores (i.e., the model predicts equally well - irrespective of high, low, and medium PSI scores of the participants).

### Importance of Variables

**Figure 1:**
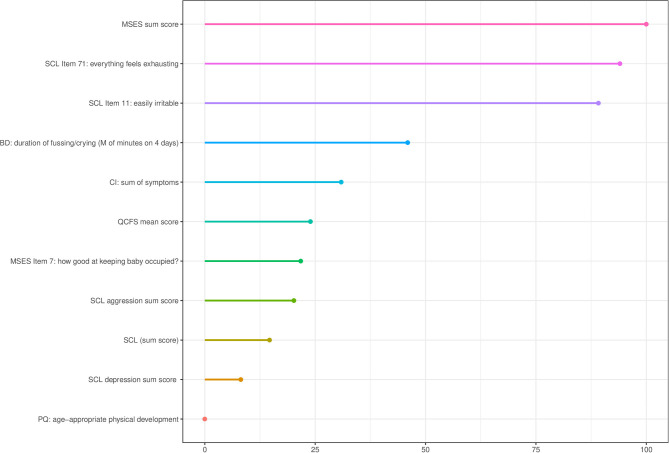
Relative importance of variables in predicting PSI extracted from the tuned model. CI, Clinical interview; MSES, Maternal Self-Efficacy Scale; QCFS, Questionnaire for Crying, Feeding, and Sleeping; SCL, Symptom-Severity-Check-List-90R-S; PQ, Parent-Questionnaire; PSI, Parenting Stress Index; BD, 96-hour behaviour diary.

Figure 1 displays the relative importance of the variables in predicting PSI. The 11 variables relatively contribute more to the prediction of mothers' MPS compared to the remaining 50 variables. After inspecting the importance graph of the final model, the most important variables were chosen since there was a large gap in variable importance compared to the rest after the top 11.

Table 2 shows the descriptive statistics of the top 11 important variables. Among the most important predictors were maternal self-efficacy (MSES sum score) and two items of the SCL-90R-S that assess exhaustion (item 71) and irritability (item 11).

**Table 2:** Descriptive statistics of the PSI outcome and the top 11 most important variables for the prediction of PSI.

**Variable**	***M* (%)**	***SD***	***Mdn***	**min**	**max**	**Range**
Outcome (PSI sum score)	131.50	31.60	131	59	218	159
BD: duration of fussing/crying (M of minutes on 4 days)	189.02	140.88	168.75	11.25	937.5	926.25
CI: sum of symptoms	12.20	5.98	12	3	35	32
MSES sum score	31.7	3.62	31	21	40	19
MSES Item 7: how good at keeping baby occupied?	2.55	0.84	3	1	4	3
PQ: age-appropriate physical development	0.97	0.24	1			2
0 (no)	6 (4.44)					
1 (yes)	127 (94.07)					
2 (uncertain)	2 (1.48)					
QCFS global score	2.21	0.22	2.22	1.63	2.83	1.21
SCL (sum score)	49.00	34.18	41	0	159	159
SCL anger–hostility subscale	4.45	4.42	3	0	20	20
SCL depression subscale	10.99	8.11	9	0	43	43
SCL Item 11: easily irritable	2.05	1.22	2	0	4	4
SCL Item 71: everything feels exhausting	1.70	1.30	2	0	4	4

*CI, clinical interview; MSES, Maternal Self-Efficacy Scale; QCFS, Questionnaire for Crying, Feeding, and Sleeping; SCL, Symptom-Severity-Check-List-90R-S; PQ, Parent-Questionnaire; PSI, Parenting Stress Index; BD, 96-h behavior diary*.

### Partial Dependency Plots

The partial dependency plots display the individual contribution of each of the 11 most important variables to the prediction of the PSI score. Additionally, we examined the interrelations of the four most important variables in predicting MPS.

**Figure 2:**
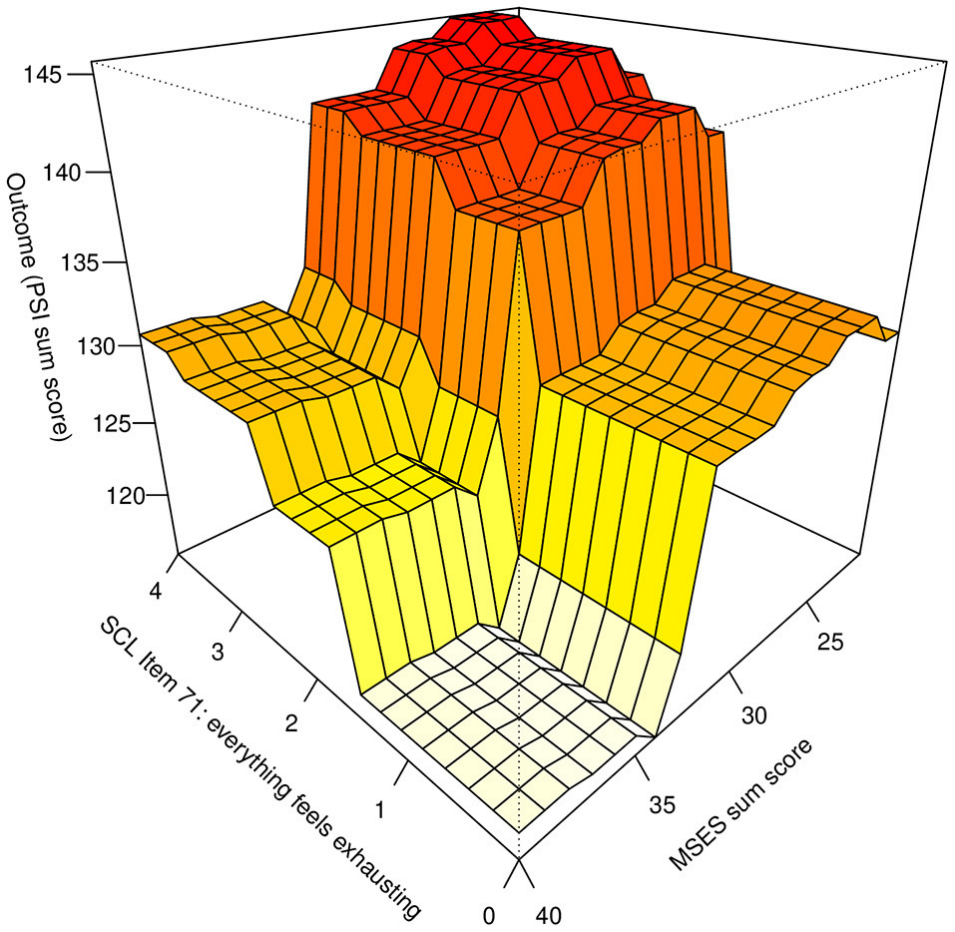
Marginal effect of MSES sum score together with the SCL Item 71 on predicted PSI value. MSES, Maternal Self-Efficacy Scale; SCL, Symptom-Severity-Check-List-90R-S; PSI, Parenting Stress Index.

**Figure 3:**
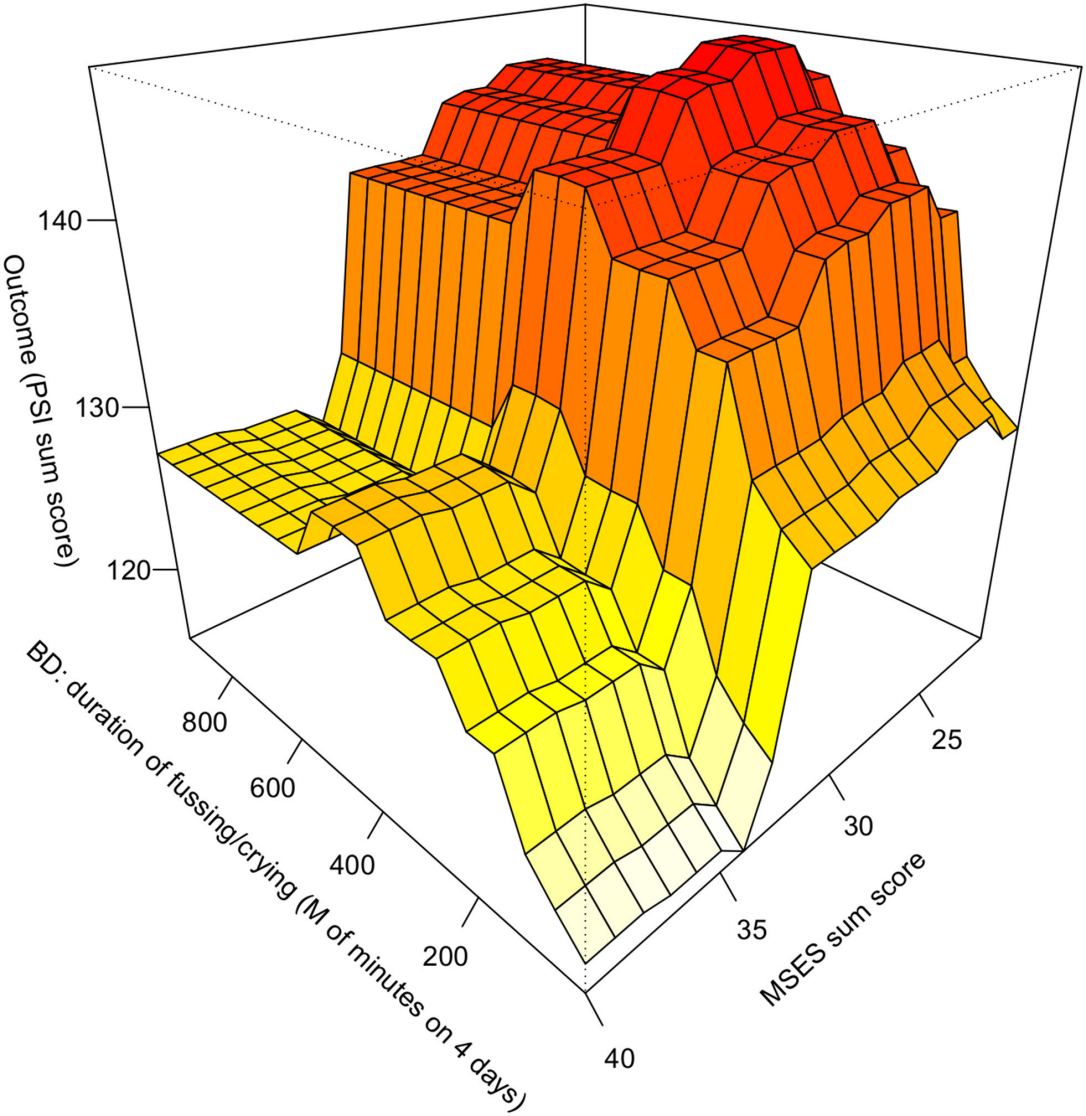
Marginal effect of MSES sum score together with the duration of fussing/crying (BD) on predicted PSI value. BD, 96-hour behaviour diary; MSES, Maternal Self-Efficacy Scale; PSI, Parenting Stress Index.

Figures 2, 3 show the marginal effect of the MSES sum score together with either item 71 of the SCL (everything feels exhausting) or the duration of fussing/crying documented in the behavioral diaries. In both figures, a plateau effect of MSES can be observed, where values lower than 31 or higher than 34 have little effect. In addition, Figure 2 shows a plateau effect for SCL-90R-S Item 71: Values below the sample mean of 1.7 are indicative of low MPS while values above 1.7 are indicative of higher PS.

Figure 3 shows a linear increasing effect of the duration of fussing/crying on MPS up until 500 min (8.33 h per day) while the plot slightly dips afterward and only five participants reported values above 500 min.

Partial dependency plots on the relation between all important variables, and the PSI score are provided in the supplement (Supplementary Figures 1–11).

## Discussion

To the best of our knowledge, this study is the first to have explored factors related to MPS in ERD by including a range of parent- and infant-related variables like behavioral, environmental, developmental, parent–infant relationship, and mental health variables. We used an ML approach as the variables of interest included a multitude of differentially scaled and potentially correlated variables that may have non-linear associations with PS and because ML enabled the use of predictor variables on single-item level in order to maximize specificity in the prediction models. As expected, mothers in our sample reported much higher average MPS compared to a normative sample, while there was a high range on the PSI score pointing to strong interindividual differences in how stressful mothers experienced parenting their child. Upon analysis of 464 variables involving self-report questionnaires, behavioral diaries, and clinical assessments, we found 11 most important predictors for MPS that can be summed up to the following areas: maternal self-efficacy, psychological distress (particularly depression and anger–hostility), infant regulatory symptoms (particularly fussing/crying), and age-appropriate physical development.

While the majority of these predictor and correlating factors support existing literature on ERD or PS in general, this study highlights their relative importance compared to other possibly relevant parent- and infant-related variables that were included and helps to specify which items particularly were relevant. Overall, our results demonstrate that a higher level of MPS in ERD was mostly associated with lower self-efficacy, stronger psychological distress symptoms particularly depression and anger symptoms, and a high degree of symptoms in the child particularly fussing and crying while the impression of the lack of an age-appropriate development was least important among the top predictors. Thus, the current psychological situation of the mother–infant dyad majorly accounted for MPS, while distal risk and protective factors were less important.

Utilizing cross-validation, we found that the model would likely generalize well to a similar population. The identified key variables can be used to select help-seeking mothers who are at an increased risk for experiencing high MPS in order to further evaluate these cases and to guide treatment of ERD. Approaching the identified factors in treatment may exert a positive effect on a mother's PS and thus on the negative reciprocities known to maintain or worsen ERD (6). Below, we discuss the important variables and implications of our results in detail.

### Maternal Self-Efficacy

The maternal self-efficacy sum score (MSES) was the most important predictor in the final model and was—as expected—negatively related to PS. The relative importance of the construct for ERD is in line with previous research: compromised maternal self-efficacy has been described as an important factor in the etiology or perpetuation of ERD (6), while higher self-efficacy may be ameliorative to PS (18). Although mothers in our sample on average rated themselves as “good enough” in terms of how effective they experienced themselves across different parenting situations, the range in this scale was broad (Table 2) with the observed minimum of 19 points being equivalent to a rating of “not good enough.” Mothers with such low expectations were prone to experience high MPS. Our research demonstrates, that in the face of ERD, mothers with low maternal self-efficacy experience high MPS and vice versa.

In addition, we identified incremental effects between low MSES scores and either exhaustion (SCL-90R-S item 71, Figure 2) or duration of infant fussing and crying (behavioral diary, Figure 3). This means for example that if a mother reported low self-efficacy in addition to experienced considerable exhaustion or experienced ≥3 h of fussing/crying per day, the model predicted significantly more MPS compared to mothers who did not fit these criteria. Thus, each of these factors combined seem to play a major role for MPS or vice versa. This result speaks for a cumulative effect where lower self-efficacy is negatively associated with MPS which gets worse the more exhausted the mother is and the more the child fusses/cries.

In addition, our results highlight a specific aspect of maternal self-efficacy important for ERD—the self-efficacy mothers experience when successfully occupying their infant: The MSES item “good at keeping baby occupied” had an additional, albeit less important role in the prediction. On average, mothers reported comparably lower self-efficacy regarding this specific parenting situation in contrast to the mean of the MSES (Table 2). This item might be especially relevant because occupying the child is a parenting task that continually arises throughout the day. Low expectations with this regard seem to be especially relevant to maternal parenting stress levels.

These results have several implications. Given the importance of maternal self-efficacy related to MPS in ERD, clinicians should assess and be aware of its deviations in order to align interventions. The combination of low self-efficacy with either high amounts of infant fussing/crying or high exhaustion of the mother should be especially considered when identifying and treating samples with ERD who target MPS. We identified a subgroup of mothers who reported high self-efficacy who experienced less MPS, despite the challenging conditions they faced. Higher self-efficacy may help in coping with prolonged fussing/crying but also in coping with exhaustion. Future research may focus on this subgroup to investigate conditions under which maternal self-efficacy can be a protective factor for MPS. This result also hints to a potential heterogeneity of etiological or maintaining factors for ERD.

### Mothers' Psychological Distress

The second area of predictors was maternal psychological distress symptoms experienced during the last week, as was reflected in the two subscales depression and anger–hostility and independently two items from these subscales (exhaustion and irritability), and in the SCL-90R-S sum score, which were all positively associated with the PSI. Surprisingly, the two single items were among the three most important predictors in the dataset. The partial dependency plots further specified nearly linear relations between mothers' exhaustion and irritability with the PSI score (see Supplementary Figures 10, 11). We noticed that mothers in our study compared to a normative sample were more psychologically distressed on average while displaying a high range on the SCL-90R-S sum score. *T*-values of the subscales depression (*T* = 60) and anger/hostility (*T* = 62) indicated a noticeable higher distress in these domains (25), suggesting that these are specific vulnerability factors in our sample.

Our results add to the notion that parents who are more depressed experience parenting in ERD as more difficult (38) and moreover specify which emotional aspect of depression is especially relevant to MPS in ERD. Accordingly, more exhausted mothers experience parenting as even more stressful compared to less exhausted mothers. It is also likely that depressive symptoms and anger–hostility inhibit parenting skills and thus increases MPS, given the studies showing that symptoms are linked to parenting impairments (39). Meanwhile, it is also plausible that a mother, who experiences more difficulties in parenting, reactively develops symptoms of depression and irritability as a result of helplessness and a lack of self-efficacy. Drawing from our results, clinical assessments and treatment conceptualization for ERD may especially consider these specific psychological distress symptoms of mothers.

While we found that current psychological distress symptoms, which in case of higher scores on the SCL-90R-S sum score point to a possible mental illness, were an important predictor for MPS, maternal lifetime mental illness was not among the critical variables. This result aligns with studies showing that PS was unrelated to prenatal anxiety or depression in no-risk infant samples (15) and may indeed play a subordinate role in parental burden related to ERD (40). However, several aspects need to be considered when interpreting our results: mothers with severe psychological distress, which increases the probability of a current acute mental illness, were excluded from study participation. Additionally, since we utilized only self-report measures, lifetime mental illness may have been underreported (41). Both of these factors may have contributed to the low prevalence rate of mental illness, thereby reducing the likelihood to generate meaningful results. Future studies should assess a more representative parent sample utilizing interview-based measures in order to clarify this question.

### Infants' Regulatory Symptoms

Three variables indicative of infants' regulatory symptoms were important in predicting PS: the duration of fussing/crying as documented by mothers in behavioral diaries, the amount of clinically assessed regulatory symptoms (sum of symptoms in the interview), and the QCFS sum score which reflects general infant regulatory symptoms severity. As expected, all variables were positively related to MPS.

Behavioral observations of prolonged fussing and crying came up as the fourth most important variable in our dataset. The importance of this variable in the prediction, as opposed to other ERD symptoms, was particularly unexpected, as only 8.9% of infants were diagnosed with persistent excessive crying disorder. Given the high prevalence rates of sleeping disorders in our sample, it would have been plausible that sleeping symptoms (e.g., number of times waking up during the night) stood out in the prediction of PSI scores. The descriptive statistics indicate an overall high level of combined fussing and crying times with a mean of over 3 h and a maximum of 15.39 h per day (Table 2). Although values >8.33 h per day were infrequent, this result is in itself an important contribution to the literature and warrants further investigation. One possible explanation for the high prevalence in our sample is that different ERD are likely related to fussing and crying. For example, difficult sleep–wake regulation has been associated with difficult temperament and low sensory thresholds, which were in turn related to increased fussing and crying (42, 43).

In our sample, there was a high comorbidity of ERD with almost 50% of the sample fulfilling diagnostic criteria of more than one diagnosis. Accordingly, the scale “sum of symptoms” covers a large range of up to 35 clinically assessed symptoms of different ERD (Table 2). As an additional predictor, the QCFS sum score, reflecting the extent of mother-reported infant overall level of crying, sleeping, and feeding symptoms and co-regulation difficulties, independently was predictive for MPS. Thus, while our results highlight the importance of infant fussing/crying for PS, we also found that the overall level of symptoms in addition is relevant to the PSI. Our results imply that for a mother in our sample, the more infant symptoms the greater the levels of MPS, irrespective of the nature and quality of the symptoms or the behavioral area affected.

Our results support previous literature on the adverse effect of prolonged crying on parents' level of perceived burden and physiological reactions in no-risk and risk samples (40, 44, 45). While it is also likely that higher MPS, which renders parents less effective in soothing their child, contributes to more regulation problems, the literature points to negative effects of dysregulation on parents (4, 5). In addition, research demonstrated that duration of fussing and crying is a precursor of maternal depression (46). It is also likely, that both factors—MPS and infants' dysregulation—exist in a reciprocal relationship with each other, thereby contributing to the perpetuation of ERD (6). Drawing from our results, the overall number of infant symptoms and especially fussing and crying related to ERD contribute to this buildup, whereas other specific infant symptoms may be less important.

These results highlight the need to utilize multiple measures in order to estimate the association between regulatory symptoms and MPS. Behavioral diaries seem to capture important aspects of everyday life that are relevant to MPS. Self-report measures may add an important subjective factor to the clinically assessed symptoms. For treatment planning, our results suggest targeting mothers' experience of prolonged and inconsolable fussing/crying in sleeping disorders and comorbid ERD.

### Infants' Age-Appropriate Physical Development

Mothers' rating of an age-adequate physical development of the child was the least important predictor in the final prediction model. While most of the mothers felt that their child was well-developed physically (94.08%, Table 2), it seems that having the impression of a “normal” development or not makes a difference to the extent of MPS. While interpreting this result, it is important to consider that infant age-appropriate developmental performance assessed with the ASQ-3 (e.g., gross-motor development), was unrelated to MPS. Thus, it seems that not the actual developmental problems but the mothers' perception thereof is what makes parenting in our sample more or less stressful. Asking mothers about their perception of infant development may be a more valuable question in order to estimate their level of MPS related to ERD.

## Limitations

While we assessed several risk factors, the use of cross-sectional assessed data in our study excludes causal data interpretation. In addition, although we included many empirically and theoretically derived variables, there are still more variables that have been shown to be related to ERD (e.g., perceived social support) and may predict MPS.

Our results' generalizability is restricted by the relatively homogeneous sample in terms of psychosocial and socio-demographic characteristics. This homogeneity led to close-to-zero variance, leading some variables to be excluded by the algorithm e.g., unemployment of one parent or both. Additionally, the exclusion criteria of this study likely limited the variance in relevant variables like organic and medical infant risk factors and maternal mental illness. While ML can handle different distributions, this has implications for generalizability. Thus, while we cross-validated all of our models within our dataset, it is likely that the final model does not generalize to unselected samples of mothers with infants presenting with ERD. For this reason, results of this study can only be generalized to similar clinical samples and will need future replication with more diverse samples and fathers.

We investigated a referred and self-referred clinical sample which restricts generalizability to mothers who do not seek help albeit experiencing the same problems with their child. However, we aimed to identify relevant predictors and correlates for MPS that may be utilized by clinicians to work more effectively and who likely see parents who seek help. Thus, our research question would not have been answered by investigating a representative sample.

Further limitations apply to some instruments used. PRFQ, MSES, and ASQ-3 are not validated in German. The Parent-Questionnaire covers some items which need further psychometric analysis (e.g., burden in pregnancy or infant development). The clinical interview utilized is not validated. However, infants' clinical characteristics in our study resemble other clinic and at-risk sample (3, 13), which speaks to the data's generalizability in this regard.

We used items in our dataset on a single-item level in order to maximize specificity and to make suggestions for future item selection. This strategy was further supported by the low reliability of some subscales (e.g., PRFQ-IC, SCL-psychoticism). However, readers should be cautious when interpreting our results not to infer an underlying construct from a single item.

## Future Research

With this study, we demonstrated that questions on the relative importance of multiple and interrelated factors in a complex field such as the one of infant mental health can be successfully investigated by utilizing ML. Based on this study, future longitudinal studies may utilize ML for the coverage of additional risk and protective factors (e.g., mental illness of both parents, social support) for PSI levels in both parents. Such investigations allow us to explore causal pathways that consider multiple infant and parent variables and their interactions within a family- and a developmentally sensitive perspective on the factors that contribute to PS in ERD. Future studies with naturalistic samples will lead to greater generalizability of the findings.

## Data Availability Statement

The raw data supporting the conclusions of this article will be made available by the authors, without undue reservation.

## Ethics Statement

The studies involving human participants were reviewed and approved by Ethical Committee Heidelberg University, Medical Faculty. The patients/participants provided their written informed consent to participate in this study.

## Author Contributions

AG and PS-P initiated this research and conceptualized the study. AG was responsible for data acquisition and data interpretation, and together with PS-P drafted the manuscript. PS-P and AG were responsible for data analysis. ST was involved in interpreting the results and drafting the manuscript. MC was involved in funding acquisition but passed away at the end of the year 2017, to the authors' great regret. All authors approved the final version of the manuscript.

## Conflict of Interest

The authors declare that the research was conducted in the absence of any commercial or financial relationships that could be construed as a potential conflict of interest.

## Publisher's Note

All claims expressed in this article are solely those of the authors and do not necessarily represent those of their affiliated organizations, or those of the publisher, the editors and the reviewers. Any product that may be evaluated in this article, or claim that may be made by its manufacturer, is not guaranteed or endorsed by the publisher.
