# Supplementary material for: Maternal Parenting Stress in the Face of Early Regulatory Disorders in Infancy: A Machine Learning Approach to Identify What Matters Most
Source: Front Psychiatry. 2021 Aug 2;12:663285. doi: 10.3389/fpsyt.2021.663285 (PMC8365191; doi:10.3389/fpsyt.2021.663285)
Supplement: Supplementary file 1 [file Data_Sheet_1.docx]

Supplementary Material

# Table 1

*Parent-Questionnaire, 96-hour behavior diary, and clinical interview: scales and items used as predictors*

| **Parent-Questionnaire (110 items, no scores)** |
| --- |
| **Areas**   1. *Sociodemographic information* (33 items)   infant (5 items): age; gender; nationality; siblings; months apart between siblings  mother/father (21 items): age; place of birth; nationality; confession; marital status; highest education; professional training; employment status  family (7 items): living conditions   1. *History of illness* (11 items)   mother/father (10 items): physical disorder; mental disorder; surgeries; accidents; number of disorders/incidents  family (relatives) (1 item): disorders/accidents/chronic diseases   1. *Recent significant life events* (5 items)   divorce/break up; loss of relative; loss of employment; financial difficulties; sudden loss of accommodation/housing   1. *Former miscarriage/abortion* (9 items)   infertility; duration of involuntary infertility; prior pre-term birth; miscarriage: week of gestation; death of a child   1. *Pregnancy* (11 items)   planned pregnancy; child desired (mother/father); degree of psychological, social, medical problems/burden during pregnancy; treatment of medical complications in hospital (yes/no; how long); usage of medication during pregnancy; smoking during pregnancy; alcohol consumption during pregnancy   1. *Obstetric history* (6 items)   week of gestation; hours in labour; type of delivery; other birth complications; subjective burden (mother/father)   1. *Infant medical history* (23 items)   weight at birth; length at birth; head circumference; incubator (yes/no; how long), artificial respiration (yes/no; how long); tube feeding (yes/no; how long); treatment of icterus; onset of problems; asked for help at another service; current treatment; medication; severe or frequent illness of the child: onset, frequency, duration of illness in days; allergies and intolerances; inpatient treatment in hospital: (yes/no), frequency, duration   1. *Infant development* (3 items)   age-appropriate physical development; age-appropriate mental development; age-appropriate social development   1. *Infant social environment* (9 items)   care provided by others: yes/no, since when, type of care, frequency, duration/length, satisfaction; change of caretaker: yes/no, number, age of child |
| **Clinical interview (150 items/scores)** |
| 1. *Past regulatory problems* (1 item)   behavioral area affected in the past (categorical)   1. *Persistent excessive crying* (6 items, 1 score)   duration of fussing/crying episodes > 3 hours per day (yes/no);  frequency of fussing/crying episodes > 3 times per way (yes/no);  fussing/crying episodes since at least 3 weeks (yes/no);  lack of success of soothing strategies (yes/no);  episodes more often during the evening (yes/no);  general burden related to persistent excessive crying (0-3);  sum of symptoms   1. *Feeding disorders (DC:0-3R)* (31 items, 6 scores)   Feeding disorder associated with concurrent medical condition (7 items, 1 score)  current medical condition associated with feeding problems (yes/no);  refusal to eat (yes/no);  more distress over course of feeding (yes/no);  fails to gain weight or loses weight (yes/no);  medical management does not fully alleviate the feeding problem (yes/no);  feeding problems since at least 2 months (yes/no);  problems in social responsivity (yes/no);  sum score  Feeding disorder associated with insults to the gastrointestinal tract (5 items, 1 score)  major aversive event or insults (yes/no);  sudden start and fast progression (yes/no);  consistent refusal (yes/no);  trigger of intense distress (yes/no);  food refusal poses an acute or long-term threat (yes/no);  sum score  Sensory food aversions (6 items, 1 score)  consistent refusal of specific foods (yes/no);  onset of food refusal during introduction of a novel type of food (yes/no);  no difficulty with preferred food (yes/no);  refusal to eat and stops eating (yes/no);  specific nutritional deficiencies (yes/no);  problems since at least 1 month (yes/no);  sum score  Infantile anorexia (5 items, 1 score)  lack of interest in food and hunger signals (yes/no);  onset while changing food (yes/no);  significant growth deficiency (yes/no);  refusal to eat adequate amounts of food (yes/no);  problems since at least 1 month (yes/no);  sum score  Feeding disorder of state regulation (4 items, 1 score)  difficulty reaching and maintaining a calm state during feeding (yes/no);  start of difficulties in newborn period (yes/no);  fails to gain weight or loses weight (yes/no);  problems since at least 2 months (yes/no);  sum score  Feeding disorder of caregiver-infant reciprocity (4 items, 1 score)  difficulties in social reciprocity while feeding (yes/no);  primary caregiver ignores feeding or growth problems (yes/no);  significant growth deficiency (yes/no);  exclusion of organic problems or developmental disorder (yes/no);  sum score   1. *Feeding disorder (AWMF)* (10 items, 1 sum score)   more than 45minutes for one feeding episode (yes/no);  less than 2 hours between feeding episodes (yes/no);  growth deficiency (yes/no);  exclusion organic disorder (yes/no);  lack of hunger signals (yes/no);  distraction or forced feeding (yes/no);  age-inappropriate eating behavior (yes/no);  rumination, vomiting (yes/no);  problems to chew, suck or swallow (yes/no);  orofacial sensitivity (yes/no);  sum score   1. *Sleep onset disorder (DC:0-3)* (5 items, 1 score)   time to fall asleep > 30 min. (yes/no);  parent stays in the room until falling asleep (yes/no);  reunions with the parent > 3 times (yes/no);  sleep onset problem episodes 5-7 times during a week (yes/no);  significant difficulties since at least 4 weeks (yes/no);  sum score   1. *Night-waking disorder (DC:0-3)* (5 items, 1 score)   time to fall asleep again > 30 min. (yes/no);  relocation to parental bed (yes/no);  frequency of night-waking during a night > 3 times (yes/no);  night-waking problem episodes 5-7 times during a week (yes/no);  significant difficulties since at least 4 weeks (yes/no);  sum score   1. *Regulation disorder of sensory processing (DC:0-3)* (31 items, 3 scores)   Hypersensitivity (16 items, 1 score)  reacts strongly to sensory stimuli (yes/no);  reacts with aversion to sensory stimuli (yes/no);  avoids strong sensory stimuli (yes/no);  difficulties with postural control and tone (yes/no);  less exploration than expected for age (yes/no);  limited sensory-motor play (yes/no);  general cautious/fearful/avoidant behavioral pattern (yes/no);  restricted range of exploration (yes/no);  fear and clinginess in new situations (yes/no);  distress when routines change (yes/no);  general avoidant behavioral pattern (yes/no);  defiant and avoidant behavior (yes/no);  negativistic behavioral pattern (yes/no);  difficulty adapting to changes in routines/plans (yes/no);  preference for repetition (yes/no);  controlling, compulsive, perfectionistic behavior (yes/no);  sum score  Hyposensitivity (8 items, 1 score)  underreacts to sensory stimuli (yes/no);  lack of responsivity in social interactions (yes/no);  restricted range of exploration (yes/no);  restricted play repertoire (yes/no);  poor motor planning and clumsiness (yes/no);  lack of interest in exploring things or in social interactions (yes/no);  fatigability (yes/no);  withdrawal from stimuli (yes/no);  sum score  Sensory stimulation-seeking/impulsive (7 items, 1 score)  craves for high-intensity sensory stimuli (yes/no);  destructive or high-risk behaviors (yes/no);  high need for motor discharge (yes/no);  impulsive and uncoordinated behavior (yes/no);  seeking constant contact with people and objects (yes/no);  recklessness (yes/no);  general high activity level (yes/no);  sum score   1. *Pervasive regulatory disorder (AWMF)* (10 items, 1 score)   additional behavioral area affected: persistent excessive crying (yes/no);  additional behavioral area affected: night-waking problems (yes/no);  additional behavioral area affected: sleep onset problems (yes/no);  additional behavioral area affected: feeding problems (yes/no);  significant difficulties since at least 4 weeks (yes/no);  significant difficulties on at least 4 days per weeks (yes/no);  symptoms vary in intensity, duration, and frequency (yes/no);  change in behavioral areas affected (yes/no);  symptoms related to specific social interaction partners (yes/no);  dysfunctional interaction patterns (yes/no);  sum score   1. *PIR-GAS score (DC:0-3R)* (1 score) 2. *Biological/organic risk scale* (10 items, 1 score)   10 items of the organic risk scale (Laucht et al., 1992)  sum score   1. *Psychosocial risk scale* (12 items, 1 score)   11 items of the psychosocial risk scale (Laucht et al., 1992)  impact of risk on the child (scale 0-3)  sum score   1. *Emotions and social functioning scale (DC:0-3R)* (4 items, 1 score)   attention and regulation (scale 1-6);  forming relationships/mutual engagement (scale 1-6);  intentional two-way communication (scale 1-6);  complex gestures and problem solving (scale 1-6);  sum score   1. *Sum scores* (7 scores)   sum of symptoms regulation disorders of sensory processing;  sum of symptoms sleep onset and night-waking disorders;  sum of symptoms feeding disorders;  sum of symptoms on axis 1 and persistence excessive crying symptoms;  sum of risk scores;  sum of symptoms on axis 1 and persistence excessive crying symptoms, risk scores, PIR-GAS, and social-emotional functioning  number of diagnosis |
| **96-hour behavior diary (139 items/scores)** |
| 1. *Items assessed at each of the 4 days* (29 items*4):   Breast feeding/feeding (minutes, frequency);  Fussing (minutes, frequency);  Crying (minutes, frequency);  Physical contact/carrying (minutes, frequency);  Sleeping during the day (minutes, frequency);  Sleeping at night (minutes);  Sleeping in separate bed (minutes);  Sleeping in parental bed (minutes);  Change of sleeping settings during the night (yes/no);  Time to fall asleep (minutes);  Parental support to fall asleep (yes/no, frequency);  Waking up during the night (frequency);  Parental support to fall asleep after waking up (yes/no, frequency);  Awake during the night (minutes);  Perceived burden related to sleeping behavior (scale 0-3);  Fusses/cries ≥3 hours (yes/no);  Applied soothing strategies (no.);  Success of soothing strategies (scale 0-2);  Duration of applying soothing strategies (minutes);  Perceived burden related fussing/crying (scale 0-3);  Fussing/crying (minutes, frequency)   1. *Scores calculated across 4 days* (23 scores):   Success of soothing strategies (scale 0-2, mean);  Applied soothing strategies (no., sum);  Perceived burden related to sleeping behavior (scale 0-3, mean);  Perceived burden related fussing/crying (scale 0-3, mean);  Parental support to fall asleep (yes/no, mean);  Parental support to fall asleep (yes/no, sum);  Parental support to fall asleep after waking up (yes/no, mean);  Parental support to fall asleep after waking up (yes/no, sum);  Time to fall asleep (minutes, mean);  Sleeping in separate bed (minutes, mean);  Sleeping in parental bed (minutes, mean);  Sleeping during the day (minutes, mean);  Sleeping during the day (frequency, mean);  Breast feeding/feeding (minutes, mean);  Breast feeding/feeding (frequency, mean);  Physical contact/carrying (frequency, mean);  Sleeping at night (minutes, mean);  Awake during the night (minutes, mean);  Waking up during the night (frequency, mean);  Duration of applying soothing strategies (minutes, mean);  Fussing/crying (minutes, mean);  Fussing/crying (frequency, mean);  Fusses/cries ≥3 hours (yes/no, sum) |

# Figures


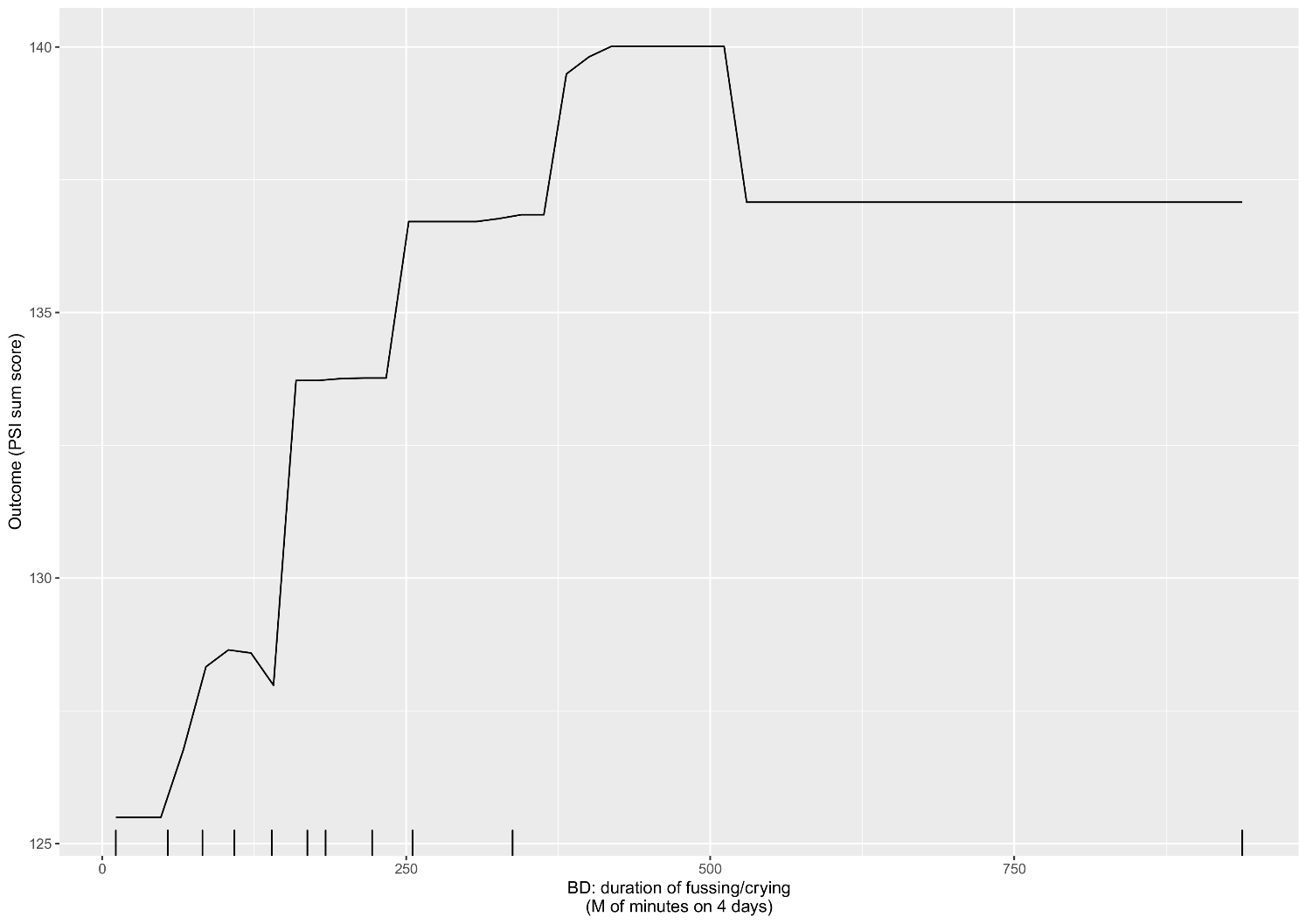


**Figure 1.** Partial dependency plot of the BD duration of fussing/crying on predicted PSI value. BD = 96-hour behaviour diary; PSI = Parenting Stress Index.


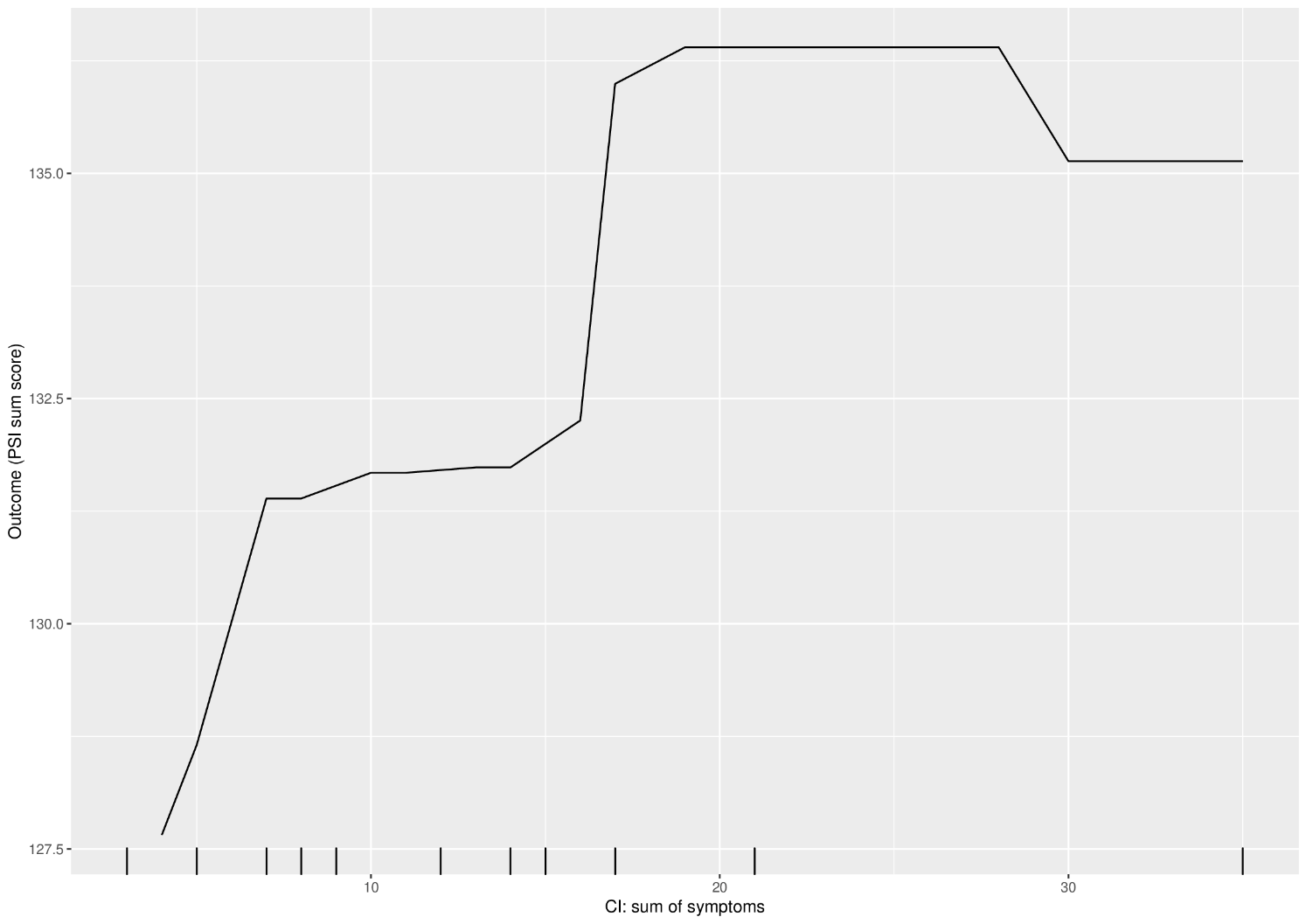


**Figure 2.** Partial dependency plot of the CI sum of symptoms score on predicted PSI value. CI = Clinical interview; PSI = Parenting Stress Index.


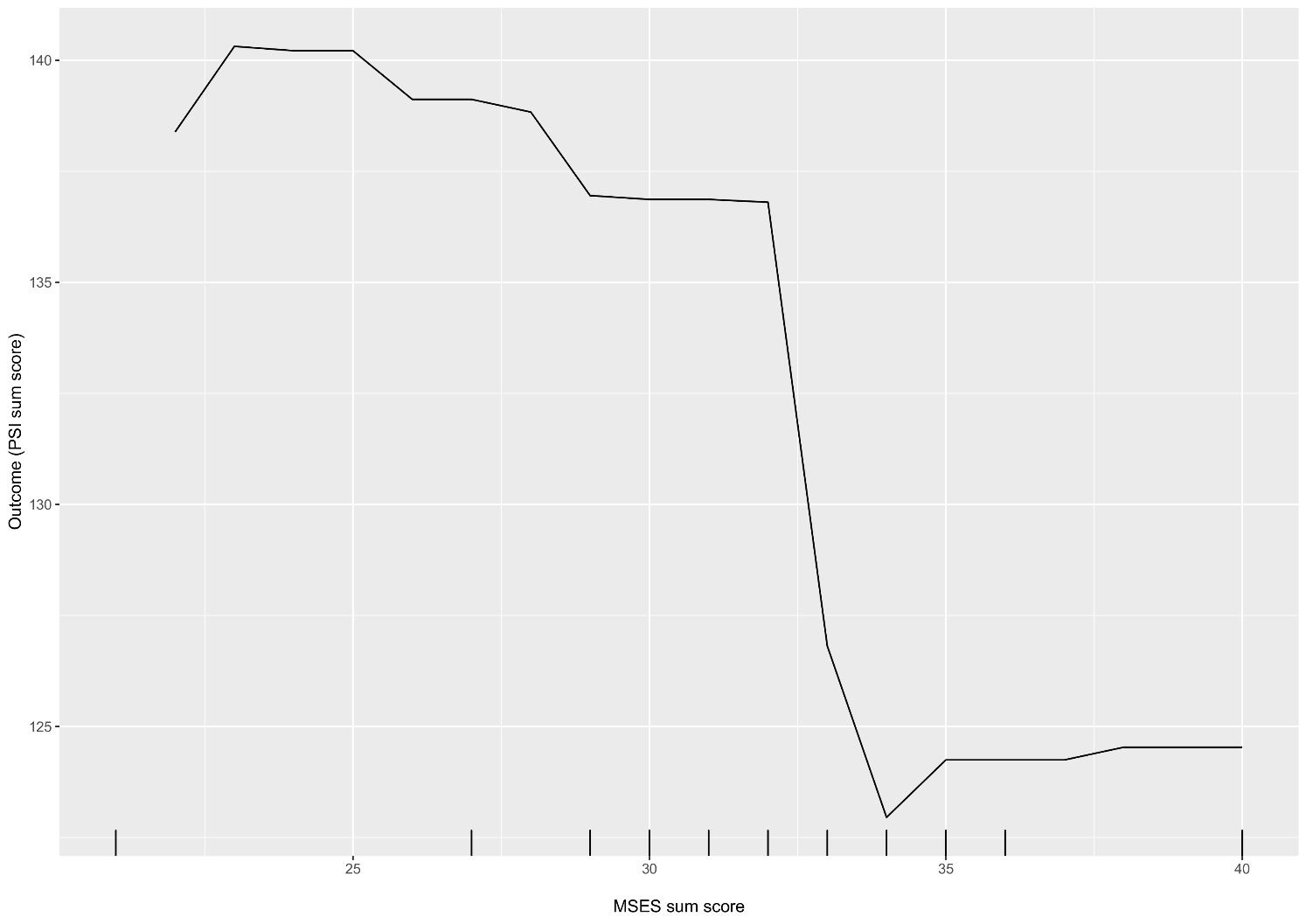


**Figure 3.** Partial dependency plot of the MSES sum score on predicted PSI value. MSES = Maternal Self-Efficacy Scale; PSI = Parenting Stress Index.


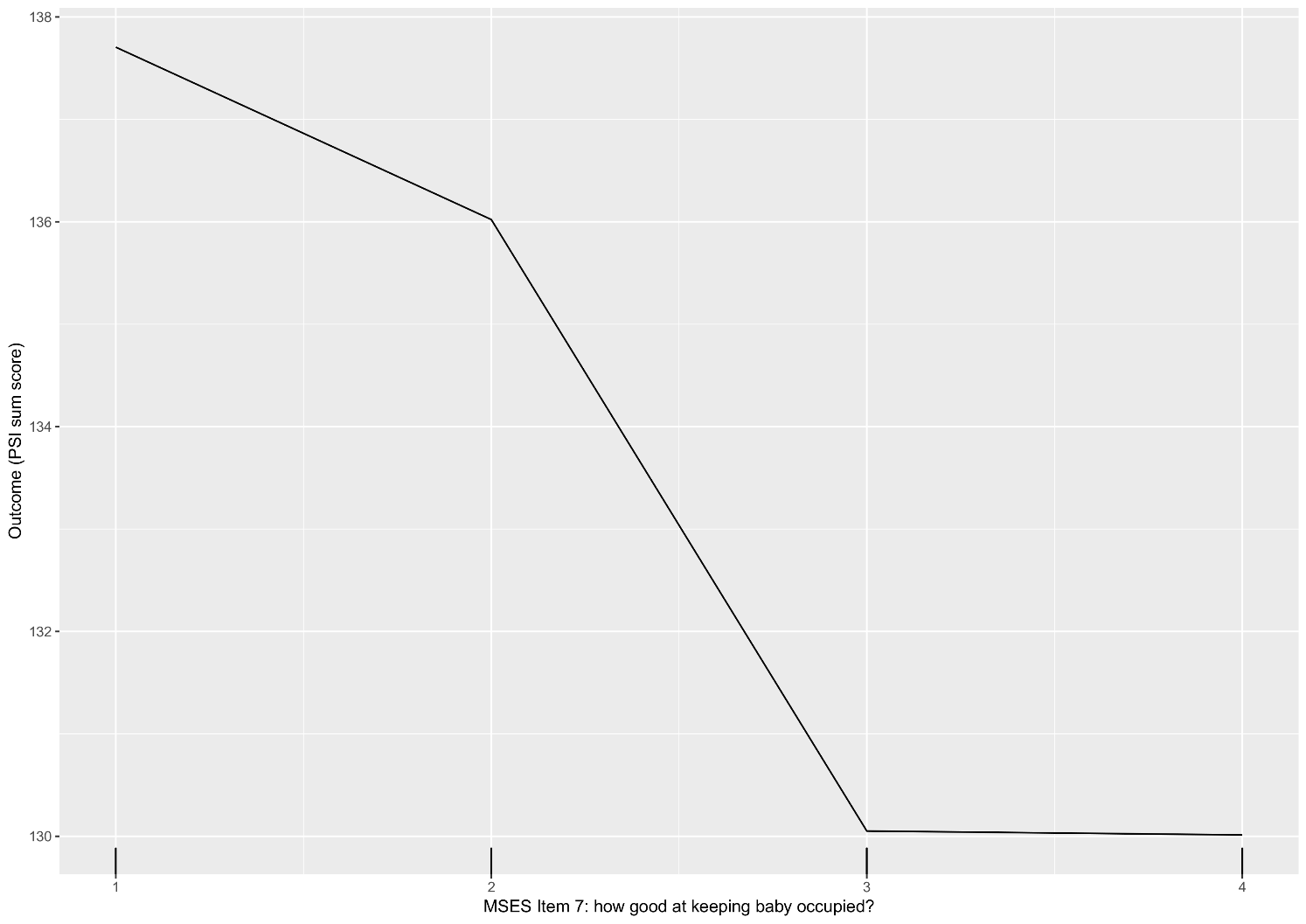


**Figure 4.** Partial dependency plot of the MSES Item 7 (how good at keeping baby occupied) on predicted PSI value. MSES = Maternal Self-Efficacy Scale; PSI = Parenting Stress Index.


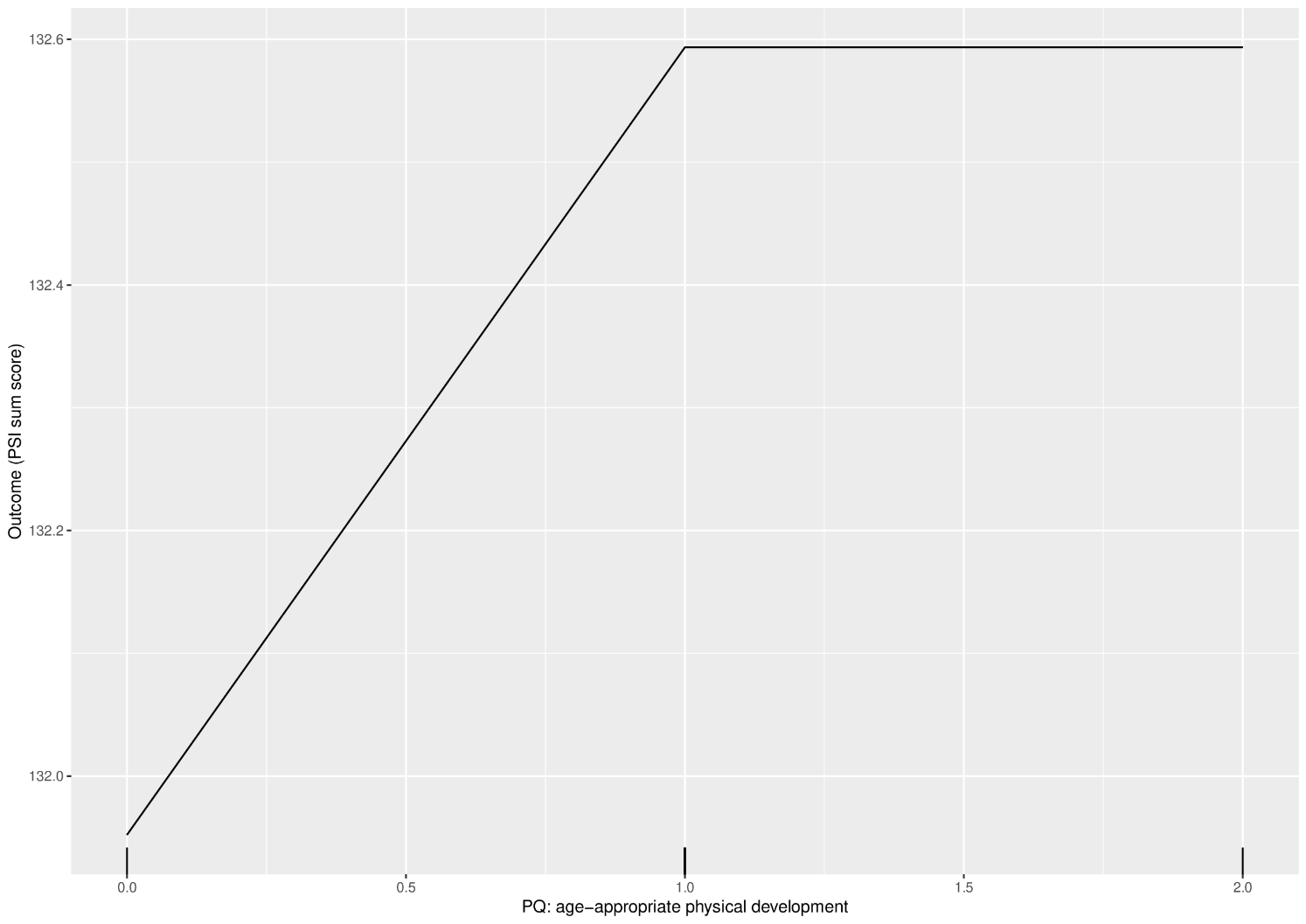


**Figure 5.** Partial dependency plot of the PQ item age-appropriate physical development on predicted PSI value. PQ = Parent-Questionnaire; PSI = Parenting Stress Index.


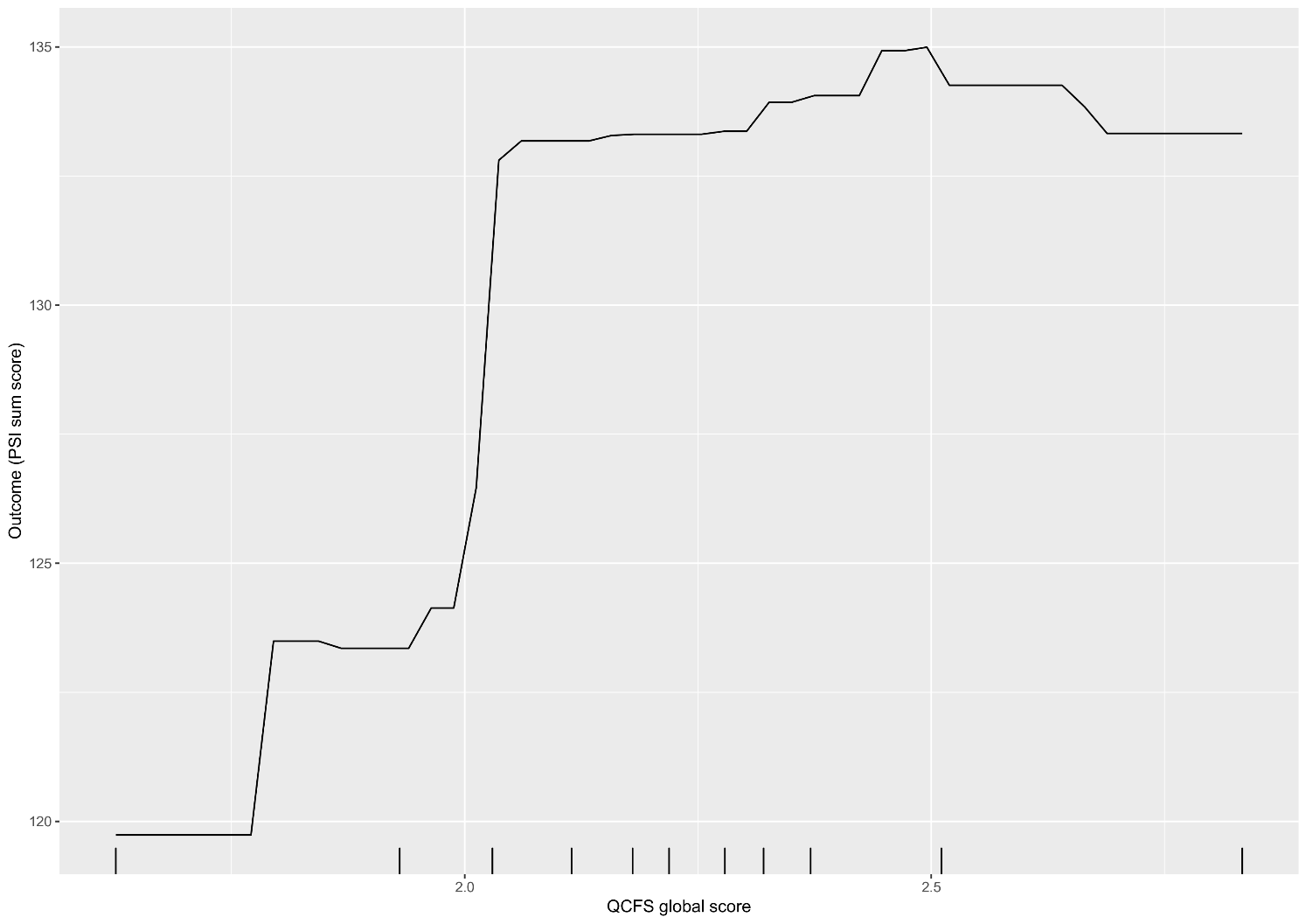


**Figure 6.** Partial dependency plot of the QCFS global score on predicted PSI value. QCFS = Questionnaire for Crying, Feeding, and Sleeping; PSI = Parenting Stress Index.


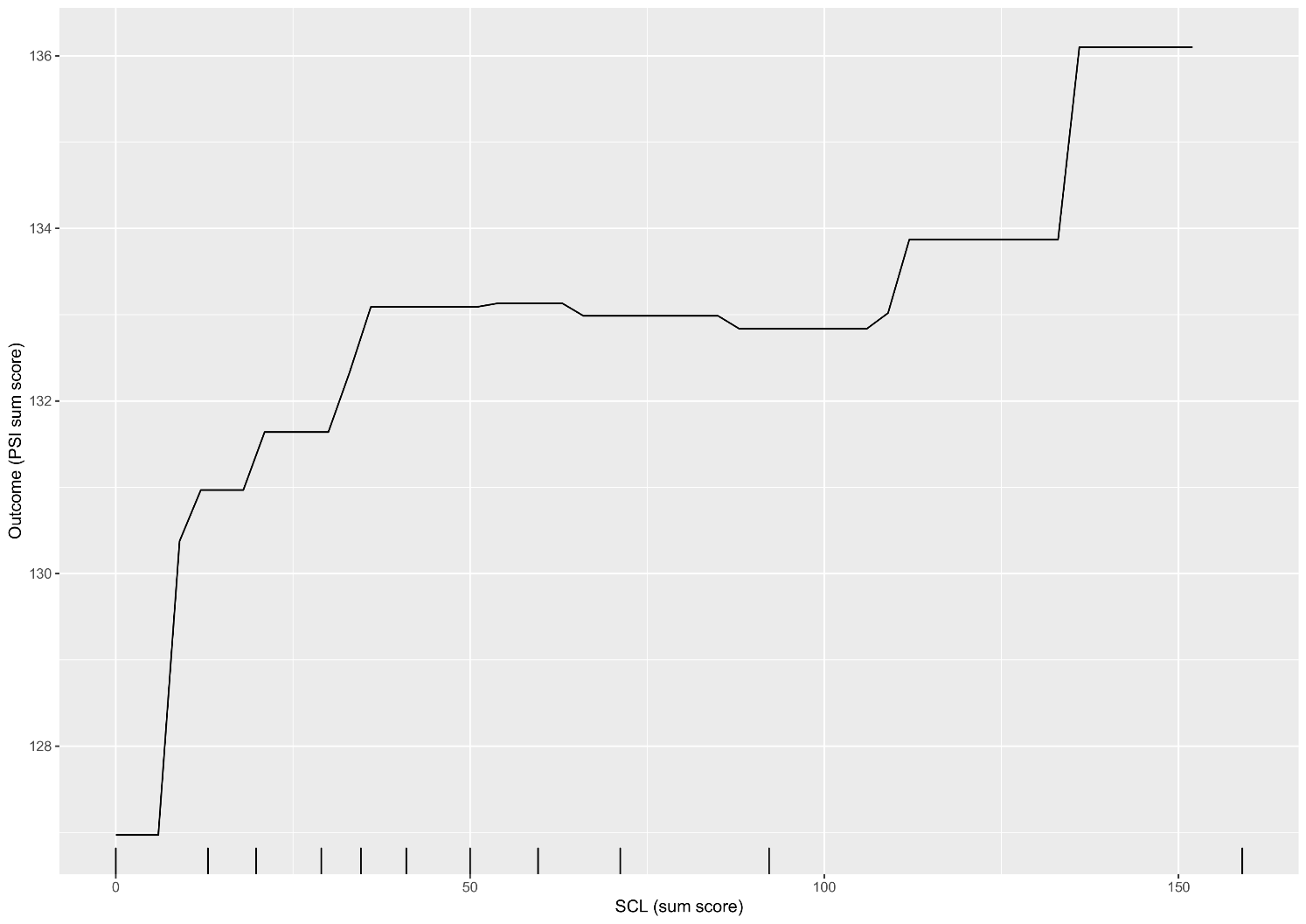


**Figure 7.** Partial dependency plot of the SCL sum score on predicted PSI value. SCL = Symptom-Severity-Check-List-90R-S; PSI = Parenting Stress Index.


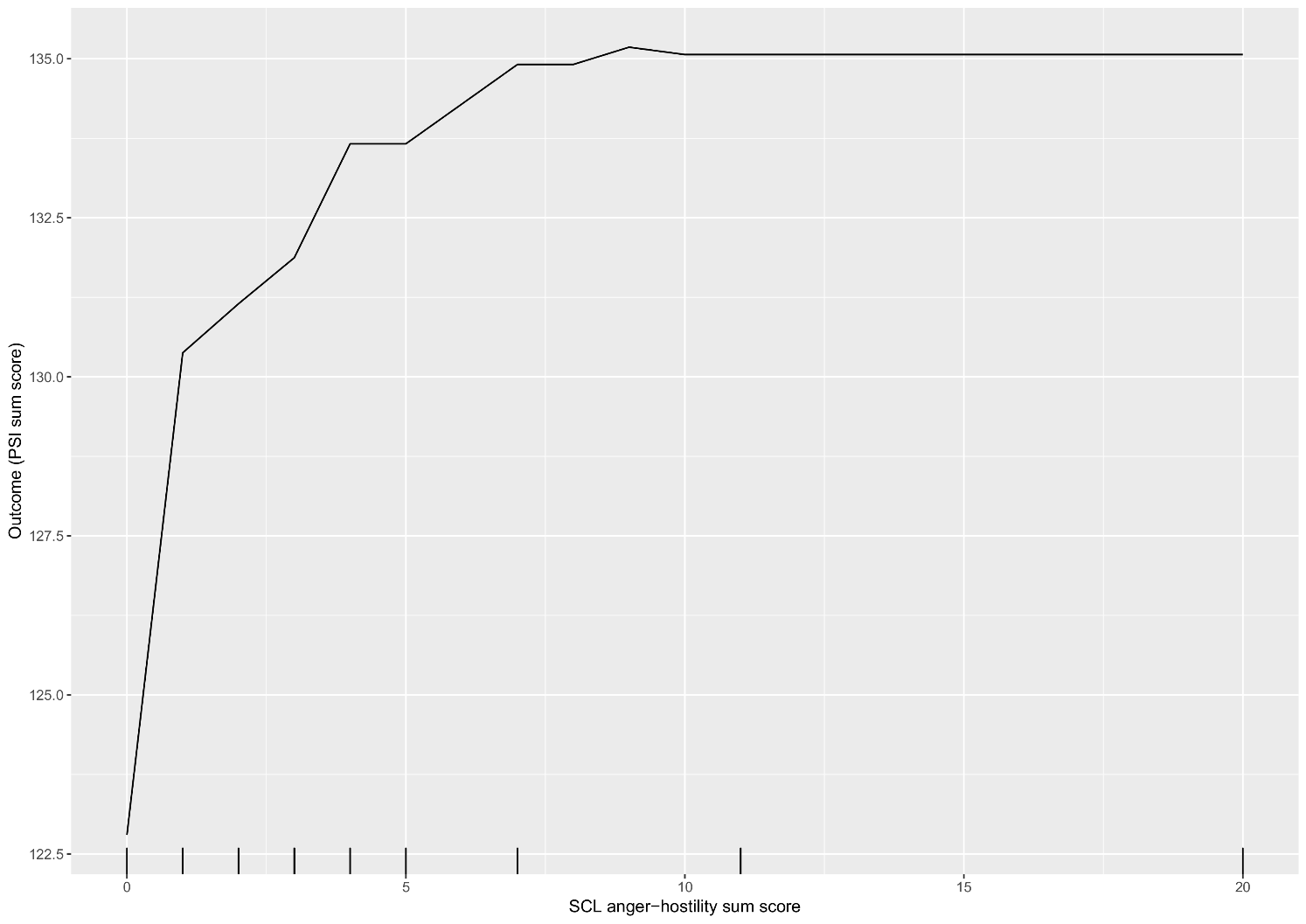


**Figure 8.** Partial dependency plot of the SCL subscale anger-hostility on predicted PSI value. SCL = Symptom-Severity-Check-List-90R-S; PSI = Parenting Stress Index.


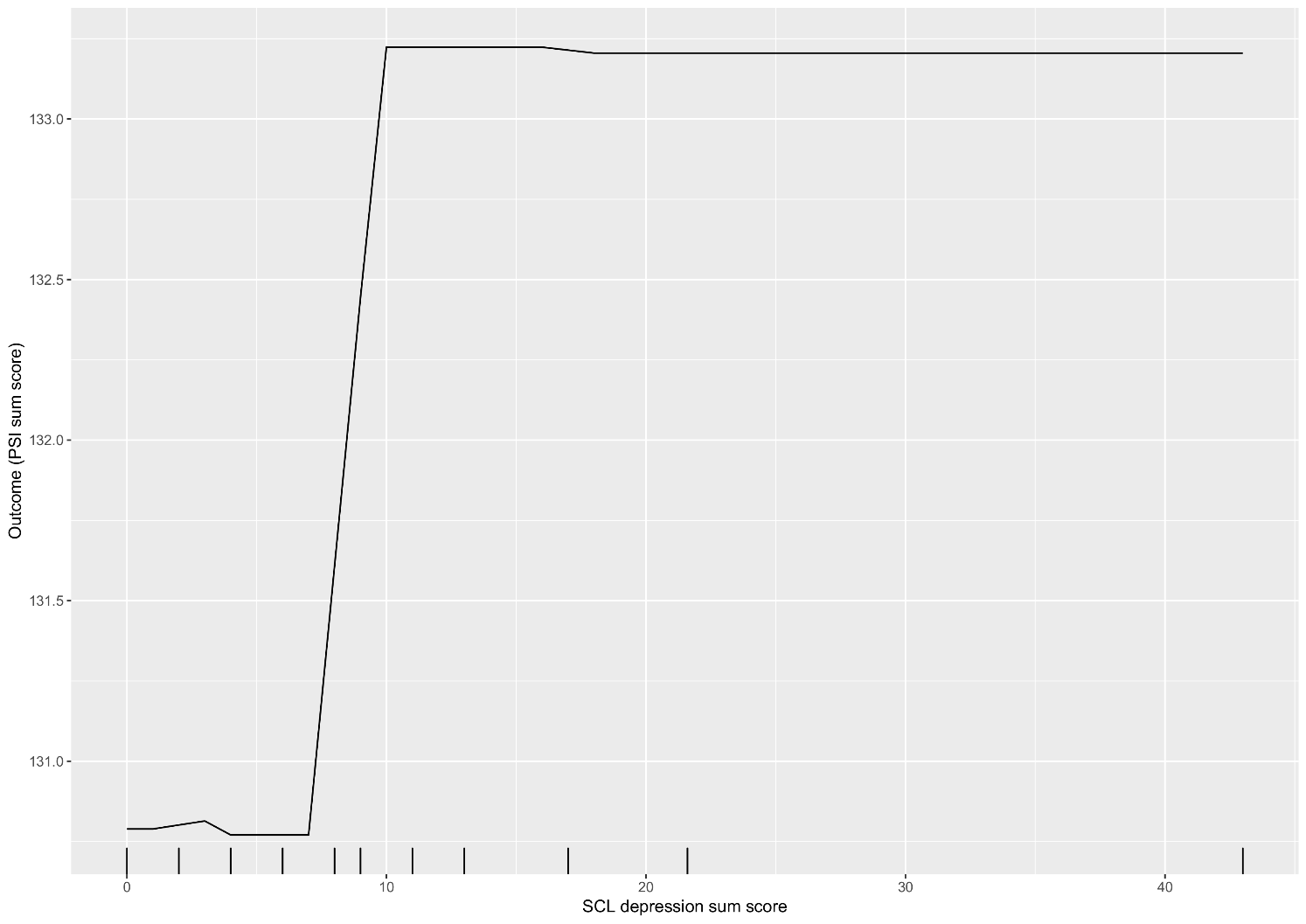


**Figure 9.** Partial dependency plot of the SCL subscale depression on predicted PSI value. SCL = Symptom-Severity-Check-List-90R-S; PSI = Parenting Stress Index.

*
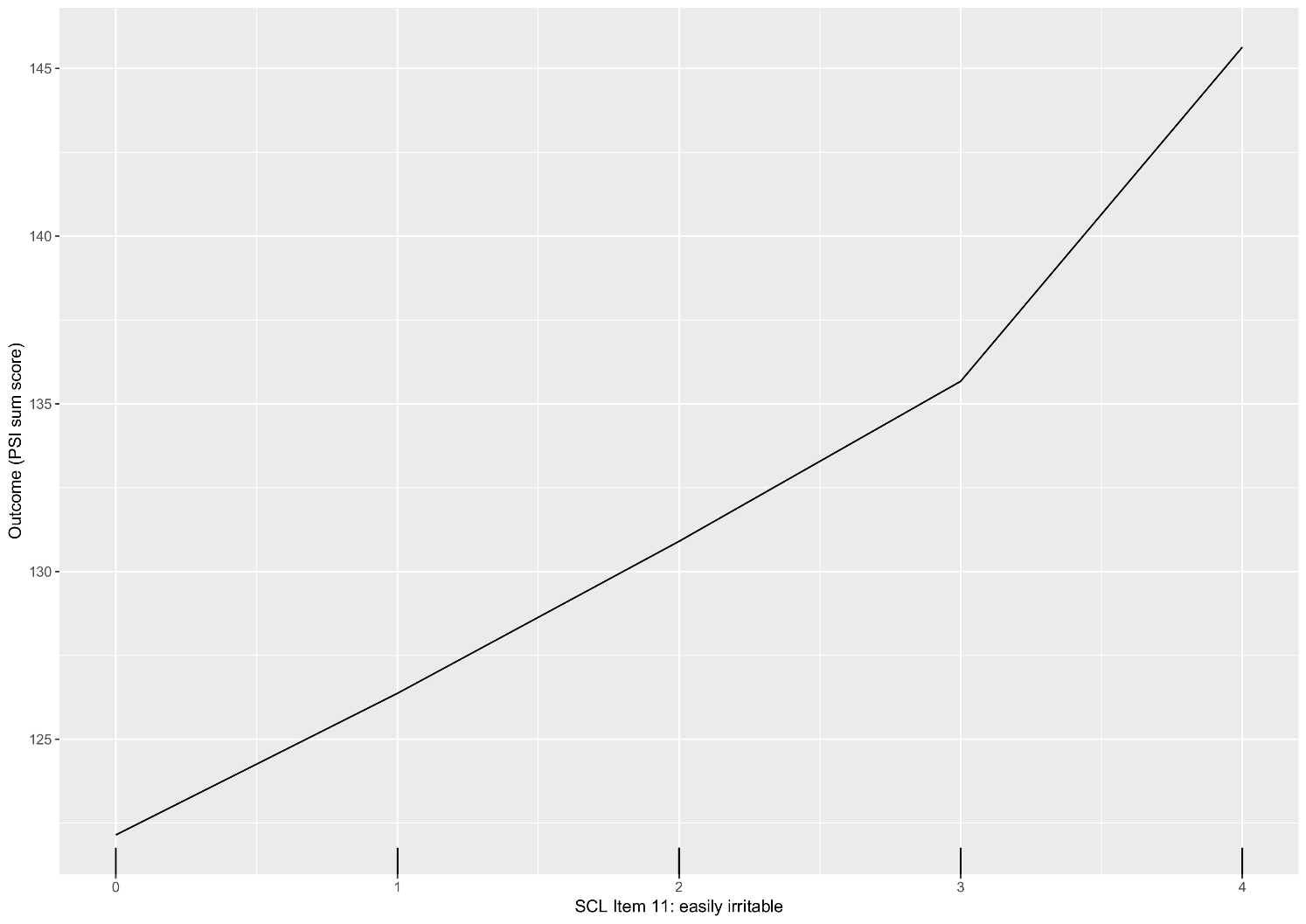
*

**Figure 10.** Partial dependency plot of the SCL Item 11 (easily irritable) on predicted PSI value. SCL = Symptom-Severity-Check-List-90R-S; PSI = Parenting Stress Index.

**
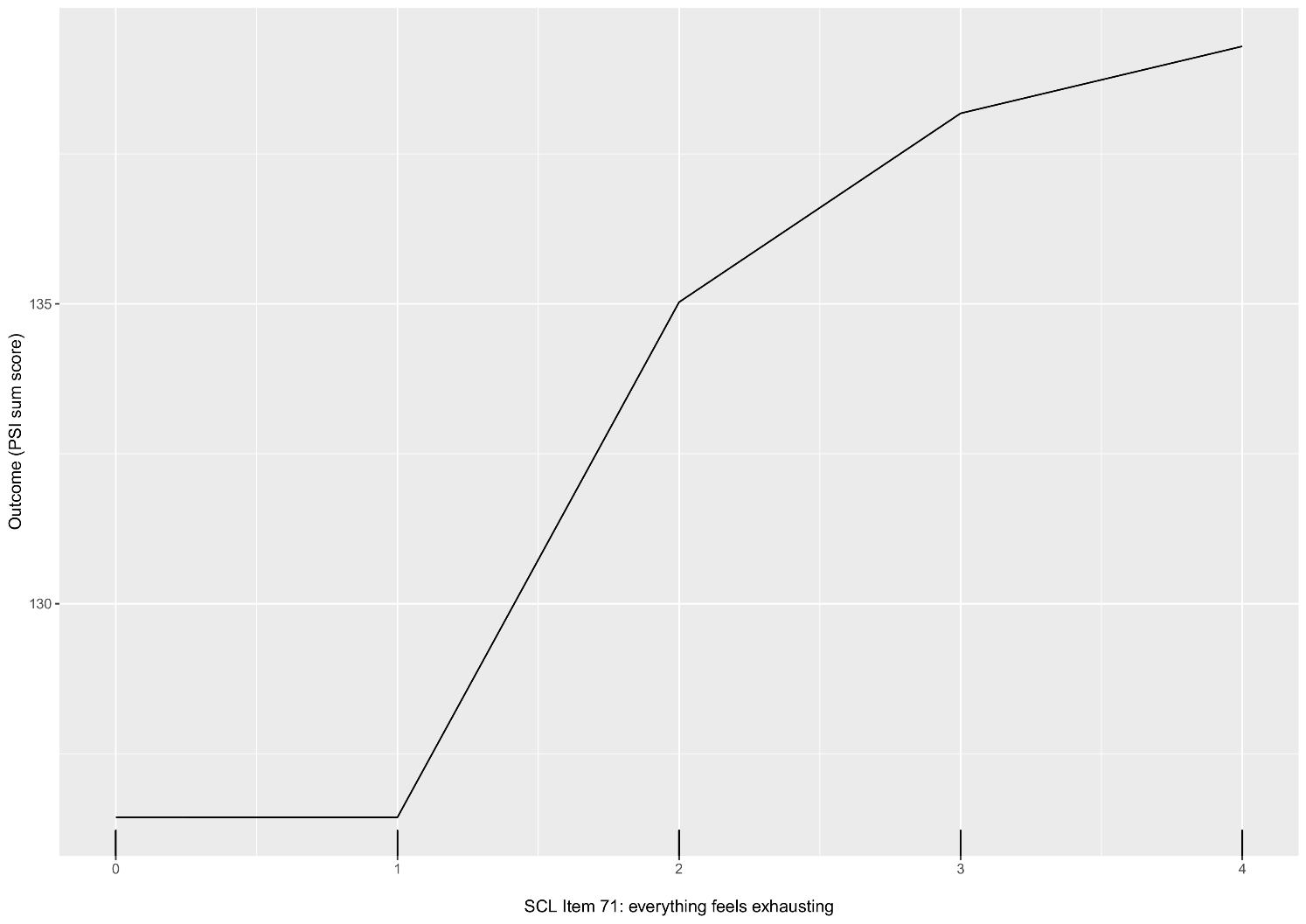
**

**Figure 11.** Partial dependency plot of the SCL Item 71 (everything feels exhausting) on predicted PSI value. SCL = Symptom-Severity-Check-List-90R-S; PSI = Parenting Stress Index.
